# Perceptions, Expectations, and Barriers to Robotic-Assisted Total Knee Arthroplasty: A Cross-Sectional Study of 218 Patients

**DOI:** 10.1055/s-0045-1812465

**Published:** 2025-11-21

**Authors:** Eduardo Frois Temponi, Álvaro Coura Castro, Renato Monteiro de Castro Junior, Rômullo Vinícius Dutra Menezes, Lúcio Honório de Carvalho Júnior

**Affiliations:** 1 Hospital Madre Teresa, Belo Horizonte, MG, Brazil; 2 Pontifícia Universidade Católica de Minas Gerais, Belo Horizonte, MG, Brazil; 3 Locomotor System Department, Faculdade de Medicina, Universidade Federal de Minas Gerais, Belo Horizonte, MG, Brazil

**Keywords:** arthroplasty, replacement, knee, health economic, robotic surgical procedures, artroplastia do joelho, economia da saúde, procedimentos cirúrgicos robóticos

## Abstract

**Objective:**

To assess the perceptions and expectations of Brazilian patients regarding robotic orthopedic knee surgery, addressing their prior knowledge, technique-related concerns, economic impact, and acceptance of the technology.

**Methods:**

The present quantitative, observational, cross-sectional study employed a structured questionnaire administered to 218 orthopedic patients undergoing knee arthroplasty at a specialized outpatient clinic. The study evaluated sociodemographic variables, information sources and levels, expectations regarding benefits, concerns related to robotic surgery, and the economic impact on decision-making. Data analysis used descriptive and inferential statistics (Chi-squared test and logistic regression), with significance set at *p* < 0.05.

**Results:**

Most patients (75%) had superficial knowledge about robotic surgery. Their chief sources of information were the attending physicians (40%) and the internet/social media (35%). In total, 60% of the patients had a positive attitude about robotic surgery, with expectations regarding greater precision (70%) and implant durability (55%). However, concerns about high costs (50%) and lack of human control (30%) were common. Willingness to pay for the procedure out of pocket was low (30%), mainly due to its high cost and lack of health insurance coverage.

**Conclusion:**

Patients showed high interest in robotic surgery, but they remained hesitant due to economic concerns and the lack of adequate information. Educational strategies and more accessible financing models can significantly increase the acceptance and adoption of this technology in Brazil.

## Resumo

**Objetivo:**

Avaliar as percepções e expectativas dos pacientes brasileiros sobre a cirurgia ortopédica robótica do joelho, abordando seu conhecimento prévio, receios relacionados à técnica, impacto econômico e aceitação da tecnologia.

**Métodos:**

Estudo transversal observacional quantitativo, utilizando questionário estruturado aplicado a 218 pacientes ortopédicos com indicação de artroplastia do joelho, atendidos em ambulatório especializado. Foram analisadas variáveis sociodemográficas, fontes e nível de informação, expectativas quanto a benefícios e receios relacionados à cirurgia robótica, além do impacto econômico na tomada de decisão. Dados analisados por estatística descritiva e inferencial (teste do Qui-quadrado e regressão logística), com significância determinada por *p* < 0,05.

**Resultados:**

A maioria dos pacientes (75%) possuía conhecimento superficial sobre a cirurgia robótica, sendo as principais fontes médicos assistentes (40%) e internet/redes sociais (35%). A cirurgia robótica foi considerada positiva por 60%, com expectativas relacionadas à maior precisão (70%) e durabilidade do implante (55%). Entretanto, preocupações sobre alto custo (50%) e falta de controle humano (30%) foram frequentes. A disposição para pagamento pessoal pelo procedimento foi baixa (30%), principalmente devido ao alto custo e à ausência de cobertura por planos de saúde.

**Conclusão:**

Os pacientes apresentam interesse elevado pela cirurgia robótica, mas permanecem hesitantes devido a receios financeiros e falta de informação adequada. Estratégias educacionais e modelos de financiamento mais acessíveis podem aumentar significativamente a aceitação e adoção dessa tecnologia no contexto brasileiro.

## Introduction

Robotic surgery has established itself as a promising innovation in orthopedics, particularly in joint reconstruction procedures such as total knee arthroplasty (TKA). This technology offers greater precision, reproducibility, and personalization in surgical planning, potentially leading to better functional outcomes and lower rates of postoperative complications. 1 2 3

Although the adoption of robotic surgery has been expanding worldwide, the literature on patients' perceptions and expectations regarding this type of procedure, especially in the preoperative period, remains scarce. 4 5 These aspects are crucial since they directly influence shared decision-making, adherence to treatment, and satisfaction with surgical outcomes. 6

No published Brazilian study systematically assessed the knowledge, fears, and barriers perceived by patients undergoing robotic arthroplasty. Considering that these perceptions often stem from information from various sources (such as doctors, the media, and social media), which is not always accurate or evidence-based, it is essential to identify potential gaps in patient understanding. 7

The present study aimed to assess Brazilian patients' knowledge, expectations, and key fears regarding robotic TKA to guide more effective doctor-patient education and communication strategies.

## Materials and Methods

The present observational, cross-sectional, analytical study had a quantitative approach. We administered a structured questionnaire ( **Appendix 1** ) to patients treated at a knee surgery outpatient clinic by a team specializing in joint replacement surgeries. The study occurred from January to May 2025. The target population consisted of orthopedic patients with indication for TKA at a center of excellence in orthopedic surgery.

We previously tested and adapted the questionnaire based on the instrument developed by Pagani et al. 6 to ensure clarity, reproducibility, and applicability in the Brazilian context.

The sample size calculation considered a universe of 500 annual robotic surgeries, a 95% confidence level, and a sampling error of 5%, resulting in a minimum number of 218 patients. The inclusion criteria were: 1) patients ≥ 18 years of age with a surgical indication for knee arthroplasty; and 2) signature of the Informed Consent Form (ICF). The exclusion criteria were: 1) patients with severe cognitive or psychiatric impairment that prevented questionnaire understanding; and 2) patients who had previously undergone robotic knee surgery.

We tabulated and analyzed the data using the IBM SPSS Statistics for Windows (IBM Corp.), version 26.0, and GraphPad Prism 9 (GraphPad Software, Inc.) software. We presented categorical variables as absolute frequencies (%), and continuous variables as means and standard deviations (SDs). Inferential analysis used the Chi-squared (χ ^2^ ) test and logistic regression, adopting a statistical significance level of *p* < 0.05.

The Research Ethics Committee approved the present study under protocol CAAE 78506523.4.0000.5127.

## Results

We organized the results into five main blocks to facilitate interpretation and statistical analysis: 1) *demographic profile* ; 2) *social issues* ; 3) *knowledge and information* ; 4) *technological preferences* ; and 5) *financial impact* and *economic barriers* .

The *demographic profile* block revealed that, among the 218 participants, the predominant age group was 55 to 70 years old (40%), followed by 40 to 54 years old (35%) and over 70 years old (25%). The sample had more males (60%) than females (40%). Most patients had low-to-moderate incomes: up to 2 minimum wages (MWs) for 50% of the patients, from 2 to 5 MWs for 35%, and above 5 MWs for 15% of participants ( Table 1 ).

**Table 1:** Knowledge and information about robotic surgery

Aspect	Category/Option	Percentage
**Surgery acceptance**	*Yes, I would accept without restrictions*	35%
	*Yes, but with reservations*	50%
	*I would not accept it*	15%
**Main concerns**	*High cost*	50%
	*Lack of human control*	30%
	*Uncertainty about safety*	20%
**Previous knowledge**	*Yes*	75%
	*No*	25%
**Information source**	*Attending physician*	40%
	*Internet and social media*	35%
	*Other patients/family members*	25%
**Understanding level**	*Good/Excellent*	30%
	*Average*	40%
	*Poor/Very poor*	30%

Regarding *social issues* , although acceptance of robotic surgery was high, half (50%) of the participants had reservations, demonstrating concerns related to the high cost (50%), the lack of human control in the procedure (30%), and safety (20%) ( Table 1 ).

In the *knowledge and information* block, 75% of participants indicated having prior knowledge about robotic surgery from attending physicians (40%) and the internet/social media (35%). However, only 30% reported having a good or excellent level of understanding of the procedure ( Table 1 ).

Regarding *technology preferences* , the perception of the benefits of robotic surgery was positive, highlighted by greater precision (70%) and higher implant durability (55%). Most patients (60%) preferred robotic surgery, while 25% remained undecided, emphasizing the need for further clarification ( Table 2 ).

**Table 2:** Technological preferences in choosing the surgical procedure

Aspect	Category/Option	Percentage
**Perceived benefits**	*Higher precision*	70%
	*Faster recovery*	45%
	*Less postoperative pain*	35%
	*Higher implant durability*	55%
**Preference between robotic and conventional surgery**	*Preference for robotic surgery*	60%
	*Undecided*	25%
	*Preference for conventional surgery*	15%

Finally, in the *financial impact and economic barriers* block, the main limiting factors were the high cost and lack of health insurance coverage. Only 45% of participants were willing to bear the costs ( Table 3 ).

**Table 3:** Financial impact and economic barriers to choosing robotic surgery

Aspect	Category/Option	Percentage
**Willingness to pay**	*I would pay out of pocket*	55%
	*I would not pay*	45%
**Factors influencing the decision**	*High cost without health insurance coverage*	55%
	*Lack of certainty about additional benefits*	35%
	*Preference for more affordable conventional alternatives*	10%

Additionally, we investigated the patients' perceptions of the robot's role in several stages of knee replacement surgery, such as incision, bone incisions, ligament balancing, implantation, and incision closure. Table 4 presents these results, providing greater clarity and detail regarding patients' opinions.

**Table 4:** Distribution of responses regarding the role of the robot in each stage of surgery

Stage of surgery	Whole procedure	Most of the procedure	Some stages of the procedure	Few stages of the procedure	I do not know
**Incision (cutting the skin down to the bone)**	15%	20%	25%	30%	10%
**Bone cuts (preparing the bone for the prosthesis)**	35%	30%	20%	10%	5%
**Ligament balancing (adjusting the tissues to balance the prosthesis)**	25%	35%	25%	10%	5%
**Implantation (fixing the prosthesis to the bone)**	20%	30%	30%	15%	5%
**Incision closure (suturing the skin and tissues)**	10%	15%	20%	40%	15%

## Discussion

### Knowledge and Perceptions about Robotic Surgery in TKA

The results of the present study revealed a significant overview of perceptions, expectations, and barriers regarding robotic TKA in Brazil. The superficial knowledge of the technique, reported by 75% of participants, is consistent with findings from Chang et al. 1 and Dandamudi et al., 2 who reported limitations in patients' understanding despite the growing interest in this technology.

The main sources of information identified in our study were the attending physicians (40%) and the internet/social media (35%). These findings were consistent with observations from Pagani et al. 6 in a public perception analysis based on crowdsourcing, in which doctors and digital media were among the main informational references.

### Expectations and Perceived Benefits

The positive perception of robotic surgery by 60% of participants, with expectations concerning greater precision (70%) and implant durability (55%), is consistent with recent literature. Studies have shown that robotic surgery is associated with less variability in component alignment, greater accuracy, and better objective and subjective outcomes compared with the conventional technique. 7 8 Recent meta-analyses confirmed that positioning accuracy and outlier reduction are consistent benefits of the robotic approach. 9 10

Although such technical gains are promising, some studies suggested that medium-term clinical benefits, such as function or patient satisfaction, do not show statistically significant differences compared with manual surgery. 11 Still, there is evidence of better early functional recovery and shorter hospital stays in patients undergoing robotic surgery. 8

### Concerns and Barriers to Adoption

The most reported concerns included the high cost (50%) and the perceived loss of human control (30%). The international literature has been documented extensively these concerns, 2 6 which are recognized as the main obstacles to the adoption of robotics, especially in public healthcare systems or those with limited coverage.

Khlopas et al. 12 also reported technological anxiety, expressed by concerns about the robot's autonomy. This anxiety appears to be related to misinformation about the collaboration role between the surgeon and the technology. However, this mistrust tends to reduce after more detailed explanations, reinforcing the importance of preoperative education. 2 13

In our study, patients expressed greater confidence in the robot for specific technical tasks, such as bone cuts (35%), while showing lower acceptance for incision closure (10%), a finding also noted by Dandamudi et al. 2

### Economic Impact and Accessibility

The low willingness to bear additional costs (45%) confirms the role of financial constraints as a structural barrier to the expansion of robotic surgery in Brazil. This finding aligns with data from studies demonstrating that most patients, especially those from lower-income brackets, are sensitive to the financial impact of this technology. 2 14

Cost-effectiveness models, as proposed by Rajan et al., 14 suggest that robotics can be cost-effective in the long term, particularly in high-volume centers, as they often lead to lower rates of complications and revisions. However, its widespread adoption in Brazil is challenging due to restricted access and limited coverage by healthcare plans.

### Comparison with Other Emerging Orthopedic Technologies

Initial resistance to robotic surgery follows a similar pattern to the introduction of other technologies, such as computerized navigation and custom implants. Studies indicated that these transitions met similar barriers, including costs, the learning curve, and uncertainty regarding clinical benefits. 15 16

### Implications for Education and Healthcare Policies

Our study found that both informational and financial barriers continue to restrict patients' acceptance of robotic surgery. The lack of knowledge about how this technology works, coupled with concerns about its safety, emphasizes the need for educational strategies promoting clear, accessible, and evidence-based information, particularly in the preoperative period.

From an institutional perspective, healthcare policies must include financing models that mitigate the effects of high costs, broaden coverage by health plans, and progressively promote the integration of robotic surgery into the public healthcare system.

Thus, the safe and equitable expansion of robotic surgery in Brazil depends on integrated actions that combine health education with the reformulation of access policies, promoting greater autonomy in decision-making and equity in the provision of technology. 17 18

### Study Limitations and Future Directions

While the present study provides relevant information on Brazilian patients' perceptions of robotic knee surgery, some limitations require consideration. First, the sample consisted of patients from a single specialized center, which may limit the generalizability of the findings to other regions of Brazil with different socioeconomic and cultural profiles. Furthermore, since this study is cross-sectional, it is not possible to assess the evolution of perceptions over time or the impact of formal educational interventions.

Another limitation concerns the self-reported nature of the questionnaire, which may be subject to recall or social desirability bias. Moreover, the lack of direct comparison with patients undergoing conventional surgery prevents a more in-depth analysis of actual differences in expectations and understanding between the groups.

Future research should utilize a longitudinal design to assess patients' perceptions both before and after educational sessions or during the postoperative period. We also recommend multicenter studies with greater geographic and demographic representation to increase the external validity of the results. In addition, the inclusion of qualitative analyses can provide deeper insights into the motivations, fears, and subjective factors that influence patients' decisions.

## Conclusion

Our study showed that, although most patients have a positive impression of robotic surgery for TKA, significant knowledge gaps and economic barriers remain, thus limiting its widespread acceptance. Most participants expressed interest in this technology, recognizing potential benefits such as increased precision and implant durability. However, concerns about the high cost, lack of health insurance coverage, and the perception of a loss of human control over the procedure were decisive factors in their hesitation. These findings emphasize the importance of tailored educational strategies, improved access to quality information, and innovative financing models to increase the availability of robotic surgery. The successful adoption of robotic surgery in Brazil will depend not only on its clinical efficacy but also on overcoming these informational and socioeconomic barriers.

## Figures and Tables

**Table:** 

	Whole procedure	Most of the procedure	Some stages of the procedure	Few stages of the procedure	I do not know
Incision (cutting the skin down to the bone)					
Bone cuts (preparing the bone for the prosthesis)					
Ligament balancing (adjusting the tissues to balance the prosthesis)					
Implantation (fixing the prosthesis to the bone)					
Incision closure (suturing the skin and tissues)					

## Notes

Appendix 1
**Patient Impressions Regarding Robotic Surgery**

You are invited to participate in the study titled “ **Patient Impressions Regarding Robotic Surgery** .” This research is being conducted by the Orthopedics and Traumatology team at Hospital Madre Teresa. The study consists of a scientific questionnaire for patients undergoing orthopedic consultations and will take approximately 5 minutes to complete. Please note that the study may be terminated early for safety or administrative reasons. Your participation in this study is voluntary and not mandatory. You may agree to participate in the study and then withdraw at any time. You can request the complete deletion of your information from this study at any time and ensure it is not used for other purposes. During the study and after its completion, when the results are published in scientific journals or presented at conferences and meetings, your identity will remain confidential. No information that could identify you publicly will be disclosed. However, during the study, people directly involved in the research may have access to your data. Even so, your data will be preserved and will not be made public. Participating in this study will not incur any additional costs to you. There will also be no payment for your participation. By entering your email address below, you agree to participate in this study. Thank you very much for your time! If you have any questions or need assistance, please contact the Research Support Team: Dr. Eduardo Frois - dreduardofroisbh@hotmail.com / 31. 3339-8455 / 31. 98369-2328
I AUTHORIZE the use of this information for the purpose of studying patients' impressions on the use of robots in joint arthroplasty (replacement surgery). The data provided will be kept confidential throughout the research process. Signature: _________________________________________________
**Robotic Surgery Impressions Questionnaire**

**NAME:** ___________________________________________________________________
**AGE:** ________________ **PHONE NUMBER:** _______________________
**SKIN COLOR:** () WHITE () BLACK () BROWN () YELLOW
**GENDER:** () Male () Female () Other
**EMAIL:** _________________________ **HEALTH INSURANCE:** __________________
**EDUCATION:** () Incomplete elementary education () Complete elementary education () Incomplete high school() Complete high school () College/University degree () Postgraduate studies
**ANNUAL INCOME:** () < R$ 2,000 () R$ 2,000–5,000 () R$ 5,000–10,000 () R$ 10,000–20,000 () > R$ 20,000

1. Have you heard about the use of robotic technology for joint prosthetics (surgical treatment of knee and hip prostheses/arthroplasty)?
  A. Yes
  B. No
2. How would you describe your general understanding of the use of robotics in joint replacement (prosthetic) surgery?
  A. Excellent
  B. Good
  C. Average
  D. Poor
  E. Very poor
3. If you have heard about robotic-assisted technology in joint replacement surgery, what were your sources? (Circle all that apply)
  A. Television
  B. Family/friend
  C. Patient information leaflets at the doctor's office
  D. Internet/social media
  E. Being in the hospital or having had previous surgery
  F. I have not heard of it
4. Based on your level of knowledge about robotic joint replacement, would you want this technology to be used during your surgery if you were indicated for the procedure?
  A. Definitely yes
  B. Probably yes
  C. I am not sure
  D. Probably no
  E. Definitely no
5. What do you think are the benefits of robotic-assisted joint replacement surgery compared with conventional surgery? (Circle all that apply)
  A. Easier surgeon's work
  B. More precise implant placement than conventional surgery
  C. Better outcomes
  D. Faster recovery
  E. Ability of artificial intelligence to guide surgery based on patient characteristics
  F. Less pain after surgery
  G. Less bleeding
  H. Longer prosthesis lifespan
6. Would you prefer robotic technology to be used during your joint replacement surgery if the outcomes were the same as with non-robotic surgery?
  A. Definitely yes
  B. Probably yes
  C. I am not sure
  D. Probably no
  E. Definitely no
7. Do you think hospitals that offer robotic-assisted technology during total knee replacement surgery are better than those that do not?
  A. Much better
  B. Somewhat better
  C. About the same
  D. Somewhat worse
  E. Much worse
8. How much value do you think robotic assistance offers surgeons in decision-making during joint surgeries?
  A. Extreme value
  B. High value
  C. Medium value
  D. Limited value
  E. I am not sure
9. If your insurance does not cover robotic-assisted surgery, would you consider paying for the use of the robot out of pocket?
  A. Definitely yes
  B. Probably yes
  C. I am not sure
  D. Probably no
  E. Definitely no
10. If you answered yes to the previous question, how much would you be willing to pay out of pocket for robot-assisted surgery?
  A. Less than R $1,000
  B. From R $1,000 to R$ 5,000
  C. From R$ 5,000 to R$ 10,000
  D. From R$ 10,000 to R$ 15,000
  E. More than R$ 15,000
11. Assuming that the use of robots adds a substantial cost (paid by you or your insurance company) to joint replacement surgery, how much better would the surgical outcome have to be to justify the use of robots?
  A. Extremely better outcomes
  B. Much better outcomes
  C. Reasonably better outcomes
  D. Satisfactory outcomes
  E. I am not sure
12. If you choose to have a robotic knee replacement, which would you prefer?
  A. A robotic system that requires a computed tomography (CT) scan for planning if it is more accurate than other robotic systems
  B. A robotic system that does not require a computed tomography (CT) scan for planning, even if it is less accurate than other robotic systems
  C. A robotic system that does not require a computed tomography (CT) scan for planning if it is equally accurate as other robotic systems
  D. I would trust my surgeon's preference
13. Some robotic knee replacement systems allow the robot to cut the bone, balance the ligaments, and confirm that the implant is correctly aligned and positioned. Would you prefer these decisions to be made by who?
  A. Only the surgeon
  B. Only the robot
  C. The surgeon makes some decisions, and the robot makes some decisions
14. Some robotic knee replacement systems require computed tomography (CT) scans of the knee for preoperative planning to ensure better programming and measurements. A CT scan is known to have a higher radiation dose than a routine X-ray. This increased radiation slightly increases the risk of cancer. If you needed a CT scan for robotic surgery, would you still want a robotic-assisted joint replacement?
  A. Definitely yes
  B. Probably yes
  C. I am not sure
  D. Probably no
  E. Definitely no
15. What concerns do you have regarding robotic-assisted total knee replacement surgery compared to conventional surgery? (Circle all that apply)
  A. It makes the surgery longer
  B. The surgeon plays a smaller role in the procedure
  C. Inexperienced surgeons achieve worse results
  D. Radiation risk if computed tomography (CT) scans are used
  E. Lack of research to support the use of robotic surgery
  F. Robot malfunctions that could cause harm to you
16. What is your perception of the role the robot plays in performing the next steps of knee replacement surgery? (Mark one “✓” per question)
